# Microbial Diversity Analysis of Sediment from Nakdong River Estuary in the Republic of Korea Using 16S rRNA Gene Amplicon Sequencing

**DOI:** 10.1128/MRA.01186-18

**Published:** 2018-10-11

**Authors:** Kyunghoi Kim

**Affiliations:** a Department of Ocean Engineering, Pukyong National University, Busan, Republic of Korea; Loyola University Chicago

## Abstract

Deterioration of sediment quality has been found in the Nakdong River Estuary after large-scale reclamations. Here, I report microbial diversity in sediments of Nakdong River Estuary in the Republic of Korea based on 16S rRNA gene sequencing by next-generation sequencing (NGS) techniques. The dominant bacterial phyla were Proteobacteria, Firmicutes, Bacteroidetes, and Planctomycetes.

## Abstract (precis)

Deterioration of sediment quality has been found in the Nakdong River Estuary after large-scale reclamations. Here, I report microbial diversity in sediments of Nakdong River Estuary in the Republic of Korea based on 16S rRNA gene sequencing by next-generation sequencing (NGS) techniques.

## ANNOUNCEMENT

Nakdong River Estuary is the largest estuary in the Republic of Korea, and it has been protected by law since the 1960s to maintain a natural ecosystem because this estuary is of great value as seaweed and shellfish grounds. The estuary is a critical stopover site for migratory birds because of its location in the center of the East Asia-Australia flyway (1, 2). However, the topography of Nakdong River Estuary has been severely changed by large coastal developments, such as the constructions of the Nakdong River Estuary Barrage (1987) and Busan New Port (under construction from 1997) (3, 4). Deterioration of sediment quality was found after the reclamations, and it is important to investigate sediment conditions for preparing countermeasures (2, 5). In the present study, I analyzed the microbial diversity of the sediment from Nakdong River Estuary using 16S rRNA gene amplicon sequencing.

The sediments were sampled using an acryl pipe (diameter, 5 cm; height, 20 cm) at Jinudo (sandbar), which is located in the northern part of Nakdong River Estuary (35°4′5.7ʺ N, 128°52ʹ56.5ʺ E), in July 2017 and stored at 4°C for further analysis. The sediment properties after sampling are shown in Table 1. Total DNA was extracted from 100 mg of sediment using the PowerSoil DNA isolation kit (Qiagen) according to the manufacturer’s instructions. A 16S rRNA gene amplicon sequencing library targeting the V3 to V4 regions was amplified with the primers 337F and 805R (6) according to the manufacturer’s protocol (Illumina, San Diego, CA). The sequencing of the prepared library was performed with an Illumina MiSeq platform using 300-bp paired ends at Theragen Etex, Inc. (Suwon, Republic of Korea) and generated 832,242 raw reads. The barcode, linker, and primer sequences were removed, and the removed reads were merged with paired-end reads using FLASH v1.2.11 (7). The reads with a low quality score (average score, < 20) or shorter than 300 bp were filtered out. The number of operational taxonomic units (OTUs) was determined by *de novo* clustering the sequences by a 97% sequence identity cutoff using QIIME software (v1.8.0) (8). Taxonomic abundance was calculated with RDP Classifier v1.1 using a confidence threshold of 0.8 (9).

**TABLE 1:** Sediment properties analyzed after sampling

Parameter	Value	SD
pH	7.00	0.07
Loss on ignition (%)	3.13	0.12
PO_4_-P (mg/liter)	0.92	0.12
NO_2_-N (mg/liter)	ND^*a*^	
ORP (mV)	−218.1	18.7
Water content (%)	38.4	0.9
NH_3_-N (mg/liter)	1.24	0.07
NO_3_-N (mg/liter)	0.02	0

a ND, not detected.

The taxonomic classifications at the phylum level were as follows: *Proteobacteria* (37.3%), *Firmicutes* (7.2%), *Bacteroidetes* (6.5%), *Planctomycetes* (5.3%), *Chlamydiae* (2.8%), *Cyanobacteria* (2.3%), *Verrucomicrobia* (1%), Arthropoda (0.7%), *Spirochaetes* (0.6%), Bacillariophyta (0.6%), and *Chloroflexi* (0.3%). This diverse microbial abundance denotes that multifaceted mechanisms are proceeding in Nakdong River Estuary. These results provide expanded knowledge of the microbial community of Nakdong River Estuary and aid in developing appropriate strategies and management tools for Nakdong River Estuary.

### Data availability.

The 16S rRNA gene amplicon sequences obtained from this study have been deposited in the Sequence Read Archive (SRA) of the NCBI database under the project accession number SRP157404.
